# Behavioral, Neurochemical and Neuroendocrine Effects of Abnormal Savda Munziq in the Chronic Stress Mice

**DOI:** 10.1155/2012/426757

**Published:** 2012-08-02

**Authors:** Nurmuhammat Amat, Parida Hoxur, Dang Ming, Aynur Matsidik, Anake Kijjoa, Halmurat Upur

**Affiliations:** ^1^Traditional Uighur Medicine Department, Xinjiang Medical University, 393 Medical University Road, Urumqi, Xinjiang 830011, China; ^2^Neurology Department, Xinjiang Medical University Affiliation of Traditional Chinese Medicine Hospital, 116 Huanghe Road, Urumqi, Xinjiang 83000, China; ^3^Instituto de Ciências Biomédicas de Abel Salazar (ICBAS), Universidade do Porto, Rua de Jorge Viterbo Ferreira 228, 40-50-313 Porto, Portugal

## Abstract

Oral administration of Abnormal Savda Munsiq (ASMq), a herbal preparation used in Traditional Uighur Medicine, was found to exert a memory-enhancing effect in the chronic stressed mice, induced by electric foot-shock. The memory improvement of the stressed mice was shown by an increase of the latency time in the step-through test and the decrease of the latency time in the Y-maze test. Treatment with ASMq was found to significantly decrease the serum levels of adrenocorticotropic hormone (ACTH), corticosterone (CORT) and *β*-endorphin (*β*-EP) as well as the brain and serum level of norepinephrine (NE). Furthermore, ASMq was able to significantly reverse the chronic stress by decreasing the brain and serum levels of the monoamine neurotransmitters dopamine (DA), 5-hydroxytryptamine (5-HT) and 3,4-dihydroxyphenylalanine (DOPAC). The results obtained from this study suggested that the memory-enhancing effect of ASMq was mediated through regulations of neurochemical and neuroendocrine systems.

## 1. Introduction

According to the Traditional Uighur Medicine (TUM), the abnormal Savda syndrome can be caused by exogenous (environmental, psychological, and emotional) as well as endogenous stimuli or stressors [1]. We thus hypothesized, in terms of modern medicine, the etiology and pathogenesis of abnormal Savda syndrome as the state under stress conditions whose symptoms, manifested in the clinical conditions of chronic diseases, include mental stress, tantrum, hypomnesis, dry skin, polydipsia, polyphagia, and memory dysfunction [2].

 Abnormal Savda Munsiq (ASMq) is a well-known complex prescription of Traditional Uighur Medicine for abnormal Savda which consists of crude drugs of ten medicinal herbs: *Adiantum capillus-veneris *L. *Alhagi pseudalhagi *(Bieb.) Desv.,* Anchusa italica *Retz., *Cordia dichotoma *G. Forst., *Euphorbia maculata *L., *Foeniculum vulgare *Mill., *Glycyrrhiza glabra *L., *Lavandula angustifolia *Mill., *Melissa officinalis *L., and *Ziziphus jujuba *Mill. ASMq is widely used in the prevention and treatment of many chronic diseases such as cancer, hypertension, diabetes mellitus, and memory dysfunction, the diseases which are associated with abnormal Savda Hilit and whose symptomatic expression is known as abnormal Savda Syndrome [3].

It is well established that the disruption of the hypothalamus-pituitary-adrenal (HPA) axis, a central pathway to the entire endocrine system, is often central to most health problems, syndromes, diseases, and even aging itself [4–6]. Hyperactive status of the HPA axis can result in increasing levels of corticotropin-releasing hormone (CRH), adrenocorticotropic hormone (ACTH), and glucocorticoids in the hypothalamus, pituitary, and adrenal cortex, respectively [7, 8]. On the one hand, stress can also alter the physiological homeostasis which can result in various neuronal, endocrine, and visceral dysfunctions [9]. Furthermore, stress is also known to alter cognitive functions, such as memory, and it has been linked to the pathophysiology of mood and anxiety disorders [7, 10]. A central feature of the stress response is the activation of the HPA axis which can result in an increase in plasma levels of glucocorticoids [11]. As a consequence of their profound effects on neurons, glucocorticoids can influence behavior, mood, and memory process [12, 13]. Neurotransmitter systems are also involved in learning and memory processes, and a substantial part of learning and memory impairments is due to changes in neurotransmission [14]. It is well established that neurotransmitters can interfere with learning acquisition and memory [15]. In this context, the memory dysfunction described in abnormal Savda syndrome could involve an excessive production of corticotropin-releasing hormone (CRH), adrenocorticotropic hormone (ACTH), and glucocorticoids in the hypothalamus, pituitary, and adrenal cortex, respectively, under the stress condition.

Although ASMq has been previously investigated pharmacologically and clinically for its antioxidant [2], immunomodulatory [3], and anticancer activities [16] as well as for its protective effects against radiation-induced and oxidative stress-induced damages [17–20], its potential protective effects against neurological hormone imbalance have never been systematically evaluated. Therefore, in our continuing effort to support the therapeutic values of Traditional Uighur Medicine (TUM), we have further evaluated the effects of ASMq on memory capability and concomitant biochemical parameters, such as neurotransmitters (ACTH, CORT, and *β*-EP), as pathophysiological indicators involved in the chronic stress mice model. The selected model is based on the fact that psychological chronic stress-induced (electric foot-shock) pathophysiology is similar to the conditions of abnormal Savda syndrome described in the ancient theory of TUM.

## 2. Materials and Methods

### 2.1. Reagents

Mice adrenocorticotropin (ACTH), corticosterone (CORT), and *β*-endorphin (*β*-EP) kits were obtained from R&D Systems, USA. Norepinephrine (NE, purity ≥ 97%), dopamine (DA, purity ≥ 99%), 5-hydroxytryptamine (5-HT, purity ≥ 99%), 3,4–dihydroxyphenylamine (DOPAC, purity ≥ 99%),and 3,4-dihydroxybenzylamine (DHBA, purity ≥ 98%) were obtained from Sigma Co., Ltd., USA. All other reagents were of analytical grade.

### 2.2. Preparation of the Aqueous Extract of ASMq

Plant materials for ASMq (Table 1) were purchased from Xinjiang Uighur Autonomous Region Traditional Uighur Medicine Hospital in Urumqi (China) and authenticated by Pharmacist Abuduwar of the Uighur Medicine Preparation Center of Xinjiang Autonomous Region Traditional Uighur Medicine Hospital. The voucher specimens (070818) were deposited at the herbarium of this Preparation Center. Briefly, all the herbs were chopped into small pieces and ground. The powdered material (1 kg) was macerated in warm distilled water (10 L) at 80°C for 12 h. The solution was then boiled for 30 min and left to stand for 1 h before filtration. The filtrate was then concentrated to semisolid mass under reduced pressure and dried at 60°C under vacuum condition. The yield of the extract was 29.4% (w/w) of the dried plant materials. The extract was dissolved in 0.5% sodium carboxymethyl cellulose (CMC-Na) solution and administered to the mice according to the experimental dosages.

### 2.3. Animal

The pathogen-free ICR male mice weighing 18–20 g (Xinjiang Medical University Animal Center, Urumqi, China) were kept under condition of controlled temperature (24°C) and illumination (12 h light cycle) and were maintained on laboratory standard diet and water freely. The animals were divided randomly into five groups of 10 mice. The mice were kept under laboratory condition for 3 days before drug administration. The mice of normal group (Gr. I) were housed and fed in the normal condition. The chronic stress was induced in mice by application of the electric-foot shock. The stress model group (Gr. II) received the repeated electric foot-shock (volume 20–30 V, interval 0.2–0.5 s during 20 min/day) in the electric foot-shock instrument before being housed in the climatic cabinet from 8:30 am–10:30 pm (at 6°C and 25–32.8% of relative humidity). The experimental procedures were approved by the guidelines of the Animal Care and Use Committee of Xinjiang Uighur Autonomous Region. 

### 2.4. Drug Administration

0.5% Sodium carboxyl methyl cellulose (CMC-Na) solution (20 mL/kg, b.w.) was administered orally to the normal (Gr. I) and the stress model (Gr. II) mice once a day during 14 days. Another groups of mice received ASMq solution orally at the dose of 2.53 g/kg (Gr. IIIa), 5.06 g/kg (Gr. IIIb), and 10.12 g/kg (Gr. IIIc), respectively, between 7:30 am–9:30 am daily during 14 days. These doses were calculated according to the conversion table of equivalent effective dose ratios from human to animals based on the body surface area. Food was withdrawn from the animals 2 h prior to drug administration but water was allowed freely. The ASMq pretreatment groups (Gr. IIIa, IIIb, and IIIc) received the same electric foot-shock one hour after drug administration (8:30 am–10:30 am).

### 2.5. Determination of Memory Capacity by the Y-Maze Task and the Passive Avoidance Task

#### 2.5.1. Y-Maze Task

The Y-maze task test was performed as previously described by Munck et al. [21]. The Y-maze apparatus with a conductive grid floor consisted of three identical arms (40 L × 10 w × 20 h cm) made of dark opaque Plexiglas and positioned at equal angles. Arms 1 and 3 were in the non-safety zone (where shocks were administered) while arm 2 was in a safety zone (on top of which there was an insulated grid floor of 10 × 15 cm). The test was conducted in two consecutive days at the same time of the day (after 14 days of oral administration with ASMq for Gr. IIIa, IIIb, and IIIc). On the first day, each mouse was placed on the top of arm 1, and a fixed resistance shock source was connected to an automatically operated switch, and electric shocks (36 V) were applied. After shocking, the mice escaped from foot-shocks by accidentally entering the top of arm 2 and this was counted as one practice and the mice were repeatedly trained for this procedure for ten more times. After a 24 h interval, the mice were successively tested ten times and their latency time to enter the safety zone (i.e., insulated grid floor) from non-safety zone for the first time and the number of errors displayed by entering the non-safety zone within ten repetitions were recorded as learning performances.

#### 2.5.2. Passive Avoidance Task: Step-Through Test

A passive avoidance reflex apparatus was provided by Xinjiang Medical University (Urumqi, China), which was separated into a lightened chamber (11 cm × 3.2 cm) and a dark chamber (17 cm × 3.2 cm), with a connecting tunnel and copper grids on the floor. The test was conducted in two consecutive days at the same time of the day (after 14 days of oral administration with different doses of ASMq for Gr. IIIa, IIIb, and IIIc). On the first day, each mouse was placed in the illuminated compartment. Each mouse received a learning trial 24 h before the test. Mice were placed into the lightened chamber and on stepping through the tunnel into the dark chamber; they would suffer a 40 V electric stimulation (in order to condition them to stay in the lightened chamber) and escape out of the dark chamber. 24 h later, mice were placed into the lightened chamber again and the latency time of mice staying in the lightened chamber and the number of times they entered the dark chamber within 5 min were recorded to evaluate their memory capacity.

### 2.6. Measurements of Adrenocorticotropin (ACTH), Corticosterone (CORT), and *β*-Endorphin (*β*-EP)

On the last day of drug administration, the blood was collected and centrifuged at 4°C; the serum was stored at −80°C before assay. Serum levels of ACTH, CORT, and *β*-EP were determined using ELISA kit (obtained from R&D Systems). The sensitivity of the assay was 1.0 ng/mL. Intra-assay and inter-assay coefficients of variation were less than 4.85% and 6.08%, respectively. The test was performed according to the manufacturer's specification.

### 2.7. Measurements of Monoamine Neurotransmitters by HPLC-FCD

Levels of monoamine neurotransmitters (NE, DA, 5-HT, and DOPAC) in serum and brain were measured by HPLC coupled with a fluorescence detector (FCD). Mice were sacrificed immediately after exposure to the stress. Blood was sampled into EDTA-containing tubes at 10:00 am, and separated in a refrigerated centrifuge at 10,000 ×g for 10 min at 4°C. The serum was stored at −80°C until assayed. After blood collection, the brains were quickly removed, frozen in liquid nitrogen, and stored at −80°C until assayed. To determine serum monoamine neurotransmitter levels, an equal volume of 0.1 M HCl was added to the serum samples containing 200 *μ*g/mL of DHBA as an internal standard. The samples were then shaken and mixed for 1.5 min in ice water. One drop of concentrated HCl was then added to the solution and mixed in ice water for another 1.5 min and then centrifuged at 3000 rpm, 4°C for 10 min. The samples of brain tissue were homogenized in ice water solution of 0.1 M HCl. Then, 0.1 M HCl solution was added to the samples (1 *μ*L/1 mg tissue) containing 200 *μ*g/mL of DHBA as an internal standard and centrifuged at 18000 rpm, 4°C for 10 min. The samples were filtered through 0.45 *μ*m microfilters (MFS Inc., USA). Aliquots (10 *μ*L) of supernatant were injected into a reverse phase HPLC column (condition: Agilent 110180 high-voltage pump coupled to a fluorescence detector, chromatographic column ZORBAX ODB C18 4.6 mm × 150 mm × 5 mm, voltage 121 V, and wavelength 360 nm). All the brain samples were weighed on an electronic scale prior to HPLC analysis, and the results were expressed as ng of monoamine/mg of wet weight tissue.

### 2.8. Statistical Analysis

Data were expressed as the mean ± standard error of the mean (SEM). The statistical significance of the differences between the groups was analyzed by one-way analysis of variance (ANOVA) following by least significant difference (LSD) tests. All statistical calculations were performed using SPSS v13.0. Value of *P* < 0.05 was considered to be significant. 

## 3. Results

### 3.1. Effects of ASMq on Memory Capability in Stress Mice

**Figure 1:**
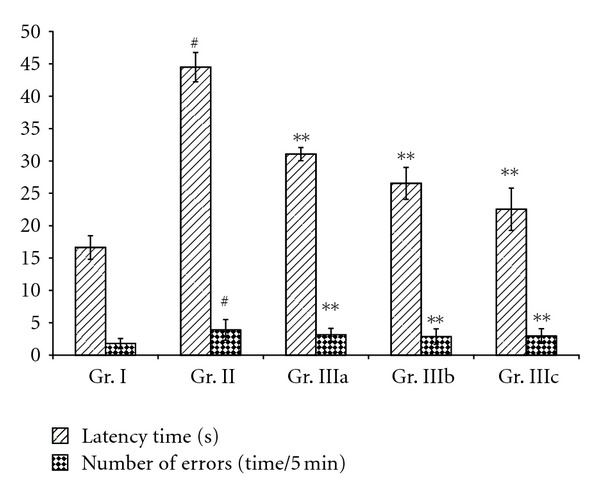
The effect of ASMq on chronic stress-induced spatial working memory deficit in the Y-maze test. According to protocol, the animals received daily administration of 2.53, 5.06, and 10.12 g/kg of ASMq for 14 days. Two weeks after the drug administration period, the Y-maze test was conducted. Each data column represents the mean ± SEM (*n* = 10). ^#^ *P* < 0.05 compared with vehicle-treated group (Gr. I). **P < 0.05 compared with vehicle-treated model group (Gr. II).

**Figure 2:**
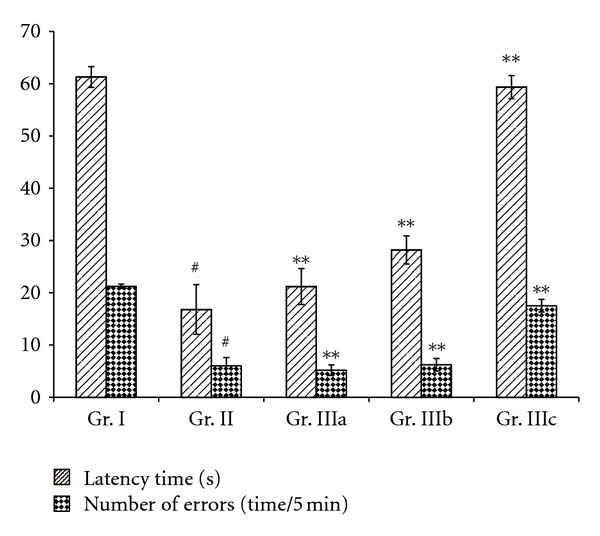
Behavioral effect of ASMq in the passive avoidance task. ASMq or vehicle was orally administered once a day for 14 days (2.53, 5.06, and 10.12 g/kg/day). The last treatment with ASMq or vehicle was administered 120 min before an acquisition trial. Twenty four hours after the acquisition trial, a 5 min retention trial was carried out. Data are expressed as means ± SEM (*n *= 10/group). ^#^ *P* < 0.05 compared with the vehicle-treated group (Gr. I). **P < 0.05 compared with the vehicle-treated model group (Gr. II).

In the Y-maze task test, the latency time and number of errors of the chronic stress mice (Gr. II) were found to be markedly increased when compared to the normal group (Gr. I). In contrast, mice treated with ASMq, by oral administration, at doses of 2.53 g/kg, 5.06 g/kg, and 10.12 g/kg (Gr. IIIa, IIIb, and IIIc) for 14 days showed an improvement of a memory as evidenced by a decrease of the latency time and the number of errors (Figure 1). On the other hand, in the passive avoidance task, the chronic stress mice (Gr. II) showed a significant decrease of the latency time and an increase in the number of errors when compared to the normal group (Gr. I). Oral administration with ASMq at doses of 2.53 g/kg, 5.06 g/kg, and 10.12 g/kg (Gr. IIIa, IIIb, and IIIc) for 14 days has boosted the memory capability as indicated by an increase of latency time and a decrease of the number of errors (Figure 2).

### 3.2. Effects of ASMq on the Serum Levels of ACTH, CORT, and *β*-EP in the Chronic Stress Mice

Table 2 showed that the serum levels of ACTH, CORT, and *β*-EP were markedly increased (*P* < 0.01) in the chronic stress mice (Gr. II) when compared to the normal group (Gr. I). Oral administration of ASMq at doses of 2.53 g/kg, 5.06 g/kg, and 10.12 g/kg (Gr. IIIa, IIIb, and IIIc) for 14 days caused a decrease of the levels of ACTH, CORT, and *β*-EP in the serum when compared to the model group (Gr. II).

### 3.3. Effects of ASMq on the Contents of Monoamine Neurotransmitters of Brain and Serum in the Chronic Stress Mice

**Figure 3:**
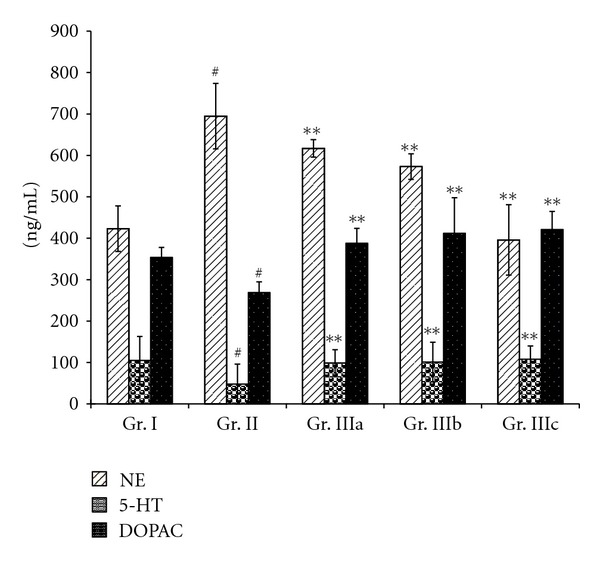
Effect of ASMq (2.53, 5.06, and 10.12 g/kg) on the concentration of NE, 5-HT, and DOPAC.

**Figure 4:**
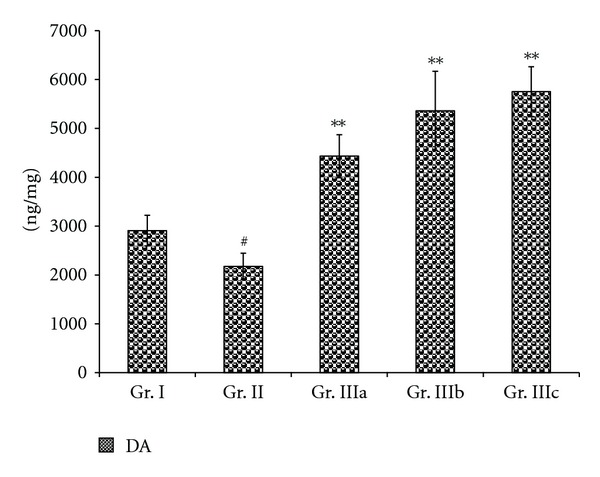
Effect of of ASMq (2.53, 5.06 and 10.12 g/kg) on the serum concentration of DA in the chronic stress. Values given are means ± SEM (*n* = 10).

Figures 3 and 4 showed an increase (*P* < 0.05) of NE level in the serum of the chronic stress mice (Gr. II) and a decrease of the serum levels of DA, 5-HT, and DOPAC (*P* < 0.01), when compared to the normal group (Gr. I). Oral administration of ASMq during 14 days at doses of 2.53 g/kg, 5.06 g/kg, and 10.12 g/kg (Gr. IIIa, Gr. IIIb, and Gr. IIIc) was able to decrease the NE levels (*P* < 0.01), while the levels of DA, 5-HT, and DOPAC were increased (*P* < 0.05). 

**Figure 5:**
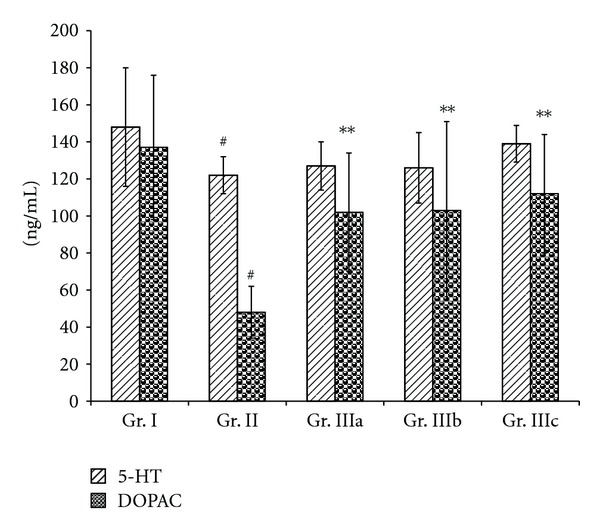
Effect of ASMq (2.53, 5.06, and 10.12 g/kg) on the concentration of 5-HT and DOPAC in the brain of the chronic stress mice. Values given are means ± SEM (*n* = 10).

**Figure 6:**
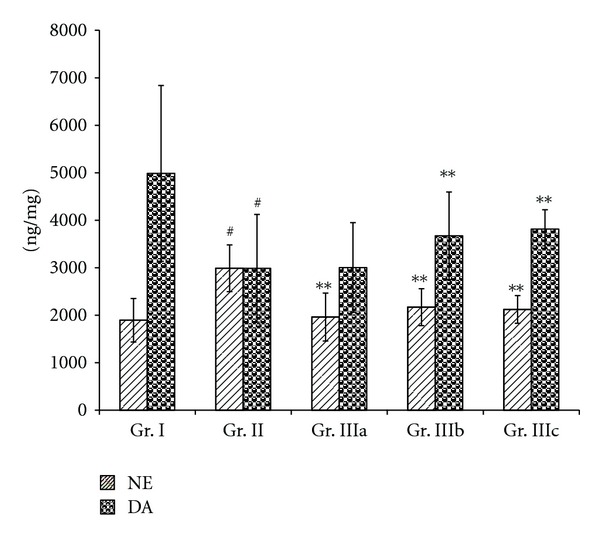
Effect of ASMq (2.53, 5.06, and 10.12 g/kg) on the concentration of NE and DA in the brain of the chronic mice. Values given are means ± SEM (*n* = 10).

Figures 5 and 6 showed similar results with an increase of the NE level (*P* < 0.01) but a decrease in the levels of DA and DOPAC (*P* < 0.01) in the brain of the chronic stress mice (Gr. II), when compared to the normal group (Gr. I). However, there were no statistically significant differences in the levels of 5-HT between the stress mice (Gr. II) and the normal group (Gr. I). All doses of ASMq (2.53 g/kg, 5.06 g/kg, and 10.12 g/kg) were found to reduce the concentration of NE in the brain (*P* < 0.01) when compared to the stress mice (Gr. II). In contrast, only the dosages of 5.06 g/kg and 10.12 g/kg of ASMq (Gr. IIIb and IIIc) could raise the levels of DA in the brain (*P* < 0.05) of the stress mice (Gr. II) while the concentrations of DOPAC in the brain were increased with all doses of ASMq (*P* < 0.05).

## 4. Discussion

It is generally accepted today that there is a strong link between stressful experiences and altered neurochemistry, endocrinology, and immunology [22], and it is also well established that unpredictable and uncontrollable stressful events can affect cognitive processes. However, only hippocampus-mediated memory processes are thought to be sensitive to the effects of chronic stress.

Many studies of nootropic (memory-enhancing) drugs use step-down, step-through, maze test, and so forth, which are often applied to the determination of capabilities of passive avoidance and spatial memory in animals to investigate their behavior changes. For this reason, we used the Y-maze task and the passive avoidance task tests to evaluate of the effects of ASMq on the memory capability of the stress mice model. Figures 1 and 2 show an improvement in learning performances of aged mice receiving ASMq by an increased latency and a decreased number of errors in the step-through test, as well as by a shortened latency and a decreased number of errors in the Y-maze test. 

In contrast, the hippocampus-independent memory processes have been shown to be resistant to chronic stressful experiences [23]. Generally, two systems are being considered in the pathogenesis of memory dysfunction—the hormones of the sympathetic nervous system (adrenalin and noradrenalin) and the hormones of the HPA axis (CRH, ACTH, and CORT). These stress-responsive systems interact at multiple levels in the periphery and in the brain, and together, they influence memory in a complex manner. Many neurotransmitters, including acetylcholine, dopamine (DA), norepinephrine (NE), and serotonin (5-HT), are found to play an important role in the learning and memory processes, and some monoamines are also known to be potent activators of the HPA axis [24]. NE is considered to be relevant to learning and memory consolidation, possibly by acting as a coordinator of signals [25]. It can also control the release of CRH in the hypothalamus and hence an ACTH mediator of the acute stress response and HPA axis, respectively. The catecholamine system, especially DA, has been also implicated in learning and memory process; however, excessive or insufficient levels of DA can lead to impairment [26]. It has been shown that DA has a beneficial impact on spatial working memory [27] and motivational processes [28] while serotonin (5-HT) may be seen also as a crucial “fine tuner” of normal and pathological processes in addition to having a role as a conventional neurotransmitter [29]. Clinical and experimental evidences have suggested that 5-HT is involved in the regulation of mood, sleep, memory, learning, and behavior, all of which are deranged to varying extents in patients with severe depression [30]. Furthermore, 5-HT has been linked to emotional processes [31], and it was found to play a particular role in emotionally related tasks. Despite the lack of functional specialization, the serotonergic system can play a significant role in learning and memory [32]. The function of HPA axis and 5-HT is intimately linked, and 5-HT is found to participate in modulating the HPA axis [33]. The results of our study have corroborated this proposal. Table 2 showed that the levels of ACTH, CORT, and *β*-EP were dramatically increased in the electric foot-shock-induced chronic stress mice and oral administration of ASMq at doses of 2.53 g/kg, 5.06 g/kg, and 10.12 g/kg for 14 days was able to reverse the levels of these hormones. Our finding is particularly relevant since it is also well known that cognitive deficits induced by various lesions to the locus are reversible by administration of drugs that enhance noradrenergic neurotransmission.

Our results showed also that oral administration of ASMq at doses of 2.53 g/kg, 5.06 g/kg, and 10.12 g/kg clearly regulated the serum and brain monoamine neurotransmitter levels after alteration induced by chronic electric foot-shock. These findings demonstrate that the brain and serum levels of NE were decreased whereas those of DA and DOPAC were markedly increased (Figures 3, 4, 5, and 6). Although the serum levels of 5-HT were found to be markedly increased, the levels of 5-HT in the brain were not statistically different (*P* > 0.05) between the model (Gr. II) and the normal groups (Gr. I).

Considering the composition of ASMq, it is pertinent to mention also that the chemical constituents of the ten herbal drugs used in the preparation could contribute to its capacity to restore memory and cognitive function of the stress mice. For example, flavonoids have been reported as constituents of *Adiantum capillus-veneris *[34], *Euphorbia maculata *[35], and *Zizyphus jujuba *[36], and emerging evidence suggested that they may exert beneficial effects on the central nervous system by protecting neurons against stress-induced injury, by suppressing neuroinflammation, and by improving cognitive function. It is likely that flavonoids exert such effects, through selective actions on different components of a number of protein kinases and lipid kinase signaling cascades, such as the phosphatidylinositol-3 kinase (PI3 K)/Akt, protein kinase C, and mitogen-activated protein kinase (MAPK) pathways rather than via their potential to act as classical antioxidants [37]. It was also reported that daily oral administration of flavonoids (35 mg/kg, for 19-20 days), isolated from aerial parts of *Scutellaria baicalensis *Georgi, could reduce memory dysfunction and neuronal injury caused by permanent global ischemia in rats [38]. Another group of phytochemicals is saponins which are constituents of *Anchusa italica *[39] and *Glycyrrhiza glabra *[40, 41]. Previous reports have demonstrated that they could be responsible for improvement of the learning impairment [42]. Zhang et al. [42] have found that tenuifolin, extracted from *radix polygalae*, was able to significantly enhance learning performances in aged mice by the step-down and Y-maze tasks. They have suggested that the improvement of learning and memory of aged mice was mediated by the effects of tenuifolin on the three stages of memory process, that is, acquisition, consolidation, and retrieval by relatively increasing the levels of NE, DA in the hippocampus and by decreasing the activity of acetylcholine esterase in the cortex. Interestingly, essential oil, which is a major constituent of *Melissa officinalis *and *Lavandula angustifolia *[43], can also exert its function on learning and memory processes. Zhang et al. [44] have reported the ameliorating effects of essential oil from *Acori graminei rhizoma *on learning and memory in aged rat and mice. By investigating the levels of cerebral neurotransmitters, they have suggested that essential oil improved cognitive function in aged rats possibly by increasing NE, DA, and 5-HT relative levels, as well as by decreasing the activity of acetylcholine esterase in the cerebra. Not surprisingly, *Melissa officinalis,* a herbal drug of the traditional medicine in many cultures, has been attributed with memory-enhancing properties. Interestingly, the ethanol crude extract of *Melissa officinalis *leaves was shown to be able to modulate mood and cognitive performance during acute administration in healthy young volunteers [44]. Finally, the aqueous extract of *Lavandula angustifolia *flowers was found to significantly block glutamate-induced neurotoxicity [45] while the methanolic extract of the whole plant of *Foeniculum vulgare *could ameliorate the amnesic effect of scopolamine and aging-induced memory deficit in mice [46], and administration of *Foeniculum vulgare *extract was able to increase step-down latency and inhibit significantly acetylcholine esterase.

## 5. Conclusion

The results of this study have demonstrated that the effects of ASMq on the HPA axis dysfunction and memory deficits, induced by chronic stress, may be related to its modulating effects on neuroendocrine and monoamine neurotransmitters. In addition to supporting traditional claims and the previously reported antichronic disease properties, this study also suggests that ASMq may prevent HPA hyperactivity induced by chronic stress. Oral administration of ASMq to the chronic stress mice was found to significantly enhance their learning performance by the step-through and Y-maze tasks. Interestingly, these effects were found to be concomitant with the regulation of monoamine neurotransmitter levels in their serum and brain. The capacity of ASMq in regulating neurochemicals and neuroendocrine system could be attributed to the presence of particular types of phytochemicals belonging to the herbal drugs constituting the ASMq preparation.

## Figures and Tables

**Table 1:** Plants for the Uighur herbal formula of Abnormal Savda Munsiq (ASMq).

Latin name	Family	Part used	Uighur name	Chinese name
*Ad* *ia* *nt* *um* *capillus-veneris *L.	Adiantaceae	whole plant	Pirsiyavxan	Tiexianjue
*Alhagi pseudalhagi* (Bieb.) Desv.	Fabaceae	branch secretion	Kök tantak	Citang
*Anchusa italica *Retz.	Boraginaceae	whole plant	Gavziban	Niushecao
*Cordia dichotoma *G.Forst.	Boraginaceae	fruit	Serbistan	Pobumuguo
*Euphorbia maculata *L.	Euphorbiaceae	whole plant	—	—
*Foeniculum vulgare *Mill.	Apiaceae	fruit	Arpabidiyan	Xiaohuixiang
*Glycyrrhiza glabra *L.	Fabaceae	radix or rhizoma	—	—
*Lavandula angustifolia Mill.*	Lamiaceae	aerial parts	Üstihuddus	Xunyicao
*Melissa officinalis *L.	Lamiaceae	whole plant	Badrenjiboye hindi	Mifenghua
*Ziziphus jujuba *Mill.	Rhamnaceae	fruit	Qilan	Dazao

**Table 2:** Effects of ASMq on the serum level of ACTH, CORT, and *β*-EP in the stress mice.

	ACTH (pg/mL)	CORT (pg/mL)	*β*-EP (pg/mL)
Gr. I	16.01 ± 3.12	12.10 ± 4.9	154.17 ± 27.19
Gr. II	32.16 ± 4.14^∗^	29.23 ± 4.5^∗^	256.21 ± 23.12^∗^
Gr. IIIa	22.31 ± 3.89^∗∗^	19.98 ± 5.6^∗∗^	201.32 ± 34.25^∗∗^
Gr. IIIb	19.64 ± 4.21^∗∗^	21.51 ± 3.34^∗∗^	193.11 ± 19.65^∗∗^
Gr. IIIc	17.56 ± 2.84^∗∗^	21.46 ± 2.1^∗∗^	176.01 ± 20.56^∗∗^

^∗^Results are given as means ± SEM, when compared to the normal group (Gr. I), *P* < 0.01.

^∗∗^Results are given as means ± SEM, when compared to the model group (Gr. II), *P* < 0.05.
